# Health effects of diesel engine exhaust emissions exposure (DEEE) can mimic allergic asthma and rhinitis

**DOI:** 10.1186/s13223-019-0342-5

**Published:** 2019-04-29

**Authors:** Elopy Sibanda, Nancy Makaza

**Affiliations:** 1 Asthma, Allergy and Immune Dysfunction Clinic, Twin Palms Medical Centre, 113 Kwame Nkrumah Avenue, Harare, Zimbabwe; 2 Department of Pathology, Medical School, National University of Science and Technology, Bulawayo, Zimbabwe; 3 Division of Pathophysiology and Allergy Research, Medical University of Vienna, Vienna, Austria

**Keywords:** Diesel engine exhaust emissions, Occupational asthma, Lung function, ANA, anti-Ro-52, Zimbabwe

## Abstract

**Background:**

Patients presenting to Accident and Emergency (A&E) facilities with dyspnoea, coughing, wheezing and nasal blockage are presumed to have allergic asthma and/or rhinitis. Occupational asthma (OA), which has similar symptoms is rarely considered. Triggers of OA include exposure to diesel engine exhaust emissions exposure (DEEEE) that are carcinogenic. We report the case of a patient who presented to an A&E facility with asthma-like symptoms, was treated for allergic asthma. Frequent exacerbations were experienced. Upon investigations it was shown that were symptoms triggered by DEEE exposure.

**Case presentation:**

A 36-year-old female bank employee was referred for the evaluation of suspected asthma. She reported a 3-month history of symptoms suggestive of asthma and rhinitis, for which she had previously required A&E treatment. There was no history of atopy. The symptoms only occurred at work or after work. Their onset had coincided with changing offices to one located proximal to a diesel-powered electricity generator. A diagnosis of asthma had been made at the A&E facility and the appropriately used inhaled fluticasone and salbutamol provided limited relief. Skin prick testing was weakly positive for seasonal pollen and house dust mite allergens. Allergen specific IgE tests for 16 regionally relevant aeroallergens were negative. Tests to exclude connective tissue diseases were positive for the anti-Ro-52/TRIM-21 autoantibody. Baseline spirometry values were markedly reduced and bronchodilator administration showed limited reversibility, FEV1 (+ 8%), PEF (+ 5%). Following a 10-day discontinuation of work exposure, the symptoms abated and FEV1 and PEF increased by 10–14% from baseline. The recent onset of asthma, in a non-atopic adult, with workday related symptoms and improvement upon discontinuation of exposure were attributed to passive occupational exposure to DEEE. The diesel generator was relocated, a short course of inhaled fluticasone and oral prednisolone was prescribed and symptoms resolved. This is the first report of the health effects of DEEE mimicking asthma and rhinitis in Zimbabwe.

**Conclusions:**

Atypical presentations of adult onset asthma in the absence of a history of either atopy or allergen specific IgE antibody sensitization should trigger in-depth evaluation of occupational exposure in all cases including office workers. Serial monitoring of lung function values should be used for diagnostic and monitoring of the patients.

## Background

Asthma is a common, heterogenous clinical condition that includes various phenotypes [1, 2]. The cardinal symptoms include wheezing, coughing, shortness of breath and tightness of the chest. Allergic asthma commonly co-exists with rhinitis whose characteristics include nasal blockage and sneezing [3, 4]. In Zimbabwe, the commonest triggers of asthma and rhinitis are house dust mites and seasonal grass or tree pollen [5]. Patients who present with asthma and rhinitis-like symptoms are likely to be diagnosed with allergic asthma or rhinitis and treated accordingly. The symptoms of dyspnoea, coughing and rhinitis are however, not unique to allergic asthma and, as stated in the timeless adage attributed to C. Jackson (1865), “*All that wheezes is not asthma*”. Causes of asthma-like symptoms vary with age and gender. Autoimmune diseases such as systemic lupus erythematosus (SLE), systemic sclerosis (SScl.), and dermatomyositis that are more common in black women from their mid-thirties are recognised causes of wheezing [6]. Occupational lung diseases are more frequent in with occupational exposure such as grain handlers, millers, bakers, cement factory workers, truck/bus depot workers and manned toll gate operators [7]. The banking office, located in a residential neighbourhood is an unlikely red flag for potential occupational asthma. Practitioners attending to patients with asthma in Accident and Emergency (A&E) departments rarely interrogate occupational exposure. Even if these were considered, the limited availability of spirometers in these settings could be a handicap.

Herein we report a case of a bank **r**eceptionist who developed asthma symptoms following exposure to diesel engine exhaust emissions (DEEE) released by a diesel-powered office electricity generator. The number of asthma patients whose symptoms are due to exposure to DEEE is unknown. The failure to accurately identify this occupational trigger of asthma and differentiate it from atopic disease has the potential of leaving a patient with a life-long label of “asthma”, with inappropriate treatment, progressive lung damage, cardiovascular complications and potentially bladder or lung cancer [8].

In Southern Africa, power outages have become common [9, 10]. The response to the disruptive outages has been a proliferation of diesel-powered electricity generators used in households and offices, extending the population at risk of exposure to DEEE. There is limited awareness of occupational causes of respiratory diseases within the medical profession. Patients presenting to busy casualty departments or general practitioners are unlikely to be evaluated for occupational allergy. Access to specialist occupational health practitioners is limited, there is one specialist in occupational medicine and one other clinical immunologist/allergist in Zimbabwe [11]. The limited availability of specialists compromises patient outcomes as the deleterious health effects of exposure to occupational hazards including DEEEE are neither identified nor mitigated. The office worker who is the subject of this report, did not meet the standard occupational risk profile. She had no history of atopy and did not smoke cigarettes. The symptoms of recent onset “asthma” had warranted two emergency A&E visits where asthma and rhinitis were diagnosed and treatment commenced. The severity of the diseases, incessant recurrence of asthma attacks and a failure to respond to optimal asthma treatment prompted specialist referral.

As diesel generators become a common source of electricity in the region, it is pertinent that health care providers are sensitized to widen their indices of suspicion beyond the stereotypical occupational risk profiles. A careful allergy history and appropriate testing including skin prick testing and spirometry was critical in excluding atopic asthma, identifying the trigger and implementing the required cessation of exposure.

## Case presentation

A 36-year-old Zimbabwean black female who had presented to her General Practitioner with shortness of breath, coughing, wheezing, nasal blockage, sneezing and throat irritation was referred to the Asthma, Allergy and Immune Dysfunction Clinic in Harare, Zimbabwe for evaluation of suspected allergic asthma. At presentation she volunteered a 3-month history of a non-productive diurnal and nocturnal cough, shortness of breath, tightness of the chest, wheezing episodes and dyspnoea. She also reported a sore throat, sneezing and itchiness of both eyes and the palate. The symptoms were worse during working hours, but also occurred during working day nights when she had difficulty breathing and constantly cleared her throat. She had no personal or family history of asthma or rhinitis until diagnosed with asthma in the preceding 3 months. She associated the exacerbation of symptoms with exposure to strong smells and generator exhaust smoke. She was using a prescribed salbutamol inhaler 2–3 times per day and used her inhaled fluticasone daily as prescribed.

### Family history and occupational history

The patient was employed as a receptionist at the offices of a banking conglomerate whose offices were located in an affluent residential neighbourhood. She lived in the neighbourhood and had been employed at the same premises for more than 10 years. She kept no pets, never smoked cigarettes and did not drink alcohol. She had no unusual hobbies. There was no family history of atopic diseases including asthma and rhinitis.

The symptoms initially manifested in August 2015, when she was relocated to her new office. Exposure to smoke, dust and strong smells for example the diesel-powered electricity generator at the workplace increasingly aggravated the symptoms. She had twice attended an A&E Department in an “attack” and asthma was diagnosed. There was transient improvement on inhaled fluticasone and salbutamol followed by frequent and often daily exacerbations despite adherence to her prescribed medicines. The past medical history was unremarkable, specifically, she denied any previous history of shortness of breath, wheezing, nocturnal dyspnoea, sneezing, allergy, fevers, productive coughing, haemoptysis, dependent oedema, or weight loss.

### General examination

On presentation she appeared well-nourished and in no obvious distress. Routine vital signs revealed a normal temperature of 36.5 °C, the blood pressure was 147/87 mmHg, the pulse was 91 beats per minute. She weighed 65 kg, stood at 156 cm giving her a healthy body mass index (BMI) of 26.7. The jugular venous pressure was not elevated. The heart was normo-rhythmic, without murmurs, rubs, or gallops. The respiratory rate was 16 breaths/min and pulmonary auscultation was normal with no wheezing. A radiologist had reported a recent chest X-ray as normal. The abdomen was soft and non-tender with normal bowel sounds. The spleen and kidneys were not enlarged. There was no palpable lymph node enlargement. The hands were pink and warm with no clubbing.

### Investigations

With a presumptive diagnosis of asthma and rhinitis, the first order of investigations was to confirm the diagnosis and to identify potential triggering allergen sources. The Asthma Control Test (ACT™) questionnaire was administered [12]. Skin prick testing (SPT) with a panel of 14 common environmental aeroallergen was conducted using extracts from Stallergenes SA, Anthony, France. Immunoblotting for allergen specific IgE antibodies was conducted using the Euroline test kit, DP3712-1601E/DP3712-6401E, (Euroimmun Medizinische Labordiagnostika AG, Lubeck, Germany). Pulmonary function testing was performed using the MSA99 Table Top Spirometer (Beijing M&B Electronic Instruments, China). Laboratory tests included a complete blood count, urea, creatinine, electrolytes and T lymphocyte subset enumeration. In view of the respiratory deficit being not bronchodilator reversible, alternate explanations were explored. A 15-parameter panel of rheumatological autoantibodies was analysed to exclude pulmonary compromise associated with connective tissue diseases using the Euroimmun ANA Profile 3 (Euroline DL1590-1601-3G) kit from Euroimmun Medizinische Labordiagnostika AG, Lubeck, Germany.

## Results

### Skin prick tests and immunoblot test results

Skin prick testing with 14 common inhalant allergen source extracts showed weak sensitization to timothy grass pollen, tree pollen and the house dust mite (*D. pter*). The wheal diameters were 4 mm against a physiological saline control of SPT wheal of 3 mm. SPT results for horse dander, *D. farinae* and *B. tropicalis* were negative. Allergen specific IgE antibodies measured using the immunoblot assay (cut-off < 0.35 kU/L) for 16 food and 11 inhaled allergen sources was negative for all 27 allergen extracts tested, including the SPT positive tree and grass pollen and *D. pter*.

### Lung function evaluation

**Fig. 1:**
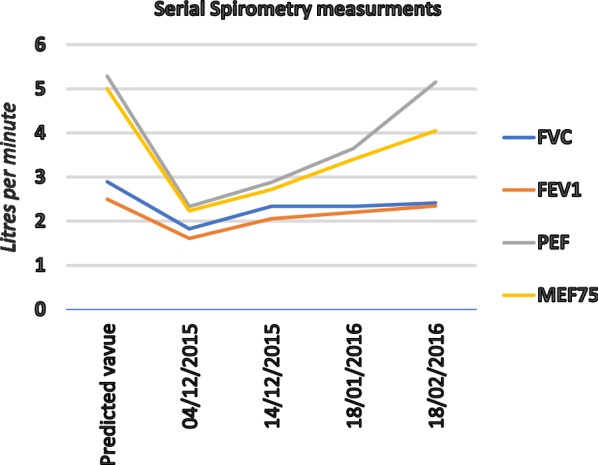
Results of serial evaluation of the patient compared to the values predicted for age, gender and ethnicity

The baseline ACT score was 18 suggesting poorly controlled asthma. The score was weighed down by frequent rescue inhaler usage and perceived poor asthma control in the preceding month. Baseline spirometry showed substantial decreases in FVC, FEV1, PEF, MEF25-75 (Table 1). With the exception of FEV 0.5, all baseline indices were significantly lower than predicted for age, ethnicity, gender and BMI. The FVC (1.83 L/min) and FEV1 (1.61 L/min) were respectively 63.1% and 64.4% while the PEF was only 44.1% of the predicted values. The PEF and MEF 75 were the lowest, being at 44.1% and 45%, respectively. Using the absolute values, the FEV1/FVC ratio was normal (88%). Nebulization with the beta-2 agonist, salbutamol, only partially reversed the deficit. The bronchodilator reversibility (FEV1 + 8%, PEF + 5%) was insufficient to conclusively demonstrate the reversible airways obstruction that is required to diagnose asthma. All parameters increased steadily upon cessation of exposure to diesel fumes, without any medication (Fig. 1).

**Table 1:** Lung function test values obtained at baseline and on three subsequent follow up visits

	Predicted (100%) values L/min	Baseline Dec. 4, 2015 (%)	Measured Dec. 14, 2015 (%)	Measured Jan. 18, 2016 (%)	Measured Feb. 18, 2016 (%)
FVC	2.91	63.1	80.7	80.7	83.4
FEV_0.5_	0.20	540.3	624.4	720.4	835.2
FEV_1.0_	2.50	64.4	82.3	85.1	93.8
FEV_3.0_	2.84	63.2	80.0	77.5	83.4
FEV1/FVC ratio	88%	88	83.7	84.7	96.4
MMF	3.02	62.7	80.1	92.9	104.4
PEF	5.29	44.1	54.5	69.0	76.7
MEF75	4.99	44.7	54.5	44.7	81.1
MEF50	3.26	68.3	77.5	68.3	107.8
MEF25	1.42	85.6	143.7	85.6	154.1

The FEV1/FVC is calculated from absolute values. The denominator (100%) is the predicted values for age, BMI and ethnicity. Values obtained following beta-2 agonist nebulisation are not shown

*FVC* full vital capacity, *FEV* forced expiratory volume in 0.5, 1.0 and 3 s (FEV_0.5_, FEV_1_, FEV_3_), *MMF* maximal mid expiratory flow, *PEF* peak expiratory flow, *MEF* mean expiratory flow, in 25, 50 and 75 s (MEF 25, 50 and 75)

### Laboratory tests

Clinical chemistry parameters were unremarkable with normal kidney and liver function parameters. The a white cell count was 6.8/mm^3^, haemoglobin (13.0 g/L), haematocrit (41.7%) and the platelet count was 280/mm^3^. The erythrocyte sedimentation rate was elevated (57 mm/h). Absolute T lymphocyte numbers were in the lower limits of the reference ranges. The total T lymphocytes count (CD3) was 836 (NR 690-2540) cells/μL, T Helper lymphocytes (CD4), 581 (NR 410-1590) cells/μL and T Suppressor/Cytotoxic lymphocytes (CD8) 262 (190–1140) cells/μL. In contrast, the lymphocyte percentages were within normal reference ranges, CD3 (60%), CD4 (42%) and CD8 (19%), thus excluding selective lymphocytopenia.

The patient sera was screened for reactivity to a panel of 15 rheumatic autoimmune disease markers, twice showing weak reactivity to the Ro-52 (TRIM 21) autoantibody. None of the remaining 14 SLE, SScl. and autoimmune inflammatory myopathy specific autoantibodies were reactive.

### Differential diagnosis

This patient had asthma and rhinitis-like symptoms that were refractory to recommended asthma therapy and punctuated by frequent exacerbations despite adherence. The ACT score was low [18] and lung volumes were significantly decreased while the FEV1/FVC ratio of 88% was normal. The co-existence of asthma-like symptoms and rhinitis was to be expected [3, 4]. The absence of a family history of atopy, unconvincing evidence of sensitization to inhaled allergen sources, poor reversibility following bronchodilator nebulization, a failure to respond to apparently appropriate asthma therapy made a diagnosis of atopic asthma tenuous. The detection of low titre anti-Ro-52 antibodies in a patient with a raised ESR (55 mm/h) and relative lymphopenia with preserved T cell percentages prompted an investigation of connective tissue diseases as potential causes or co-factors. No clinically apparent connective tissue diseases were observed.

The history was re-evaluated, carefully incorporating details of the history and occupational exposure. The patient was confident that her symptoms had only started upon changing offices 3 months previously and denied any previous episodes of asthma. Exposure to a diesel-powered electricity generator positioned some 20 m from her new office was a new development. As a front desk employee, the doors to her office were often open. The symptoms were reportedly worse at work and during evenings after working days. They were better during weekends and when away from work. She further admitted that due to frequent power outages, the stand-by diesel powered generator was used more frequently. She could not correlate power outages with symptom severity, and had never thought there could be a connection.

In view of this occupational history, a negative history of atopic allergy and an absence of convincing allergic sensitization, occupational allergy was considered. In order to investigate whether the exposure to diesel fumes at work was the trigger of the asthma and rhinitis symptoms, the patient was then removed from the work environment (sick leave) and scheduled for re-assessment after a week of non-exposure to the work environment. No medications were prescribed and she was advised to refrain from any pharmacotherapy for that duration. Upon spirometry re-testing 10 days later, lung function tests showed a spontaneous 10–14% improvement in all indices (Fig. 1, Table 1). The weak reactivity to the Ro-52 antigen persisted but could not explain the improvement of respiratory symptoms and spirometry values after a mere 10-day absence from work. In light of these findings, adverse health effects of diesel engine exhaust emissions exposure (DEEEE) was considered the trigger of her asthma and rhinitis symptoms.

### Management

Case management was guided by the assumption that DEEEE, was the trigger of the asthma and rhinitis symptoms. The first step was to limit exposure to the emissions. The employers agreed to relocate the generator away from the patient’s office. Prednisolone (30 mg once a day) for a week and inhaled fluticasone 100 micrograms twice per day and salbutamol inhaler (as required) were prescribed. Monthly follow-up visits during which spirometry measurements were performed were scheduled (Table 1). Progressive clinical and spirometry improvement was observed (Fig. 1) and the patient was discharged to the care of her family practitioner 3 months after initial presentation. Two years after the diagnosis she remains symptom free and in employment.

## Discussion

The patient presented with asthma symptoms co-existing rhinitis and pharyngeal symptoms. She experienced frequent exacerbations despite being on recommended asthma treatment. Marked reductions in all lung volumes were documented. There was suboptimal reversibility of airways obstruction following the administration of a bronchodilator. Based on the temporal association between work exposure to DEEE and the manifestations of asthma-like symptoms in the absence of allergen specific IgE aeroallergen reactivity, and cognisant of the observed cross-shift decreases in lung volumes, it was concluded that occupational exposure to DEEE had triggered the asthma-like symptoms.

### Making the diagnosis

The World Allergy Organisation position paper on occupational asthma recognises (a) asthma exacerbated by work and (b) asthma caused by work and distinguishes allergic from non-allergic irritant induced occupational asthma (OA) [13]. Algorithms recommend lung function testing for the confirmation of an asthma diagnosis. The FVC was low (63.1%) with a normal FEV1/FVC ratio (88%), a pattern associated with restrictive lung diseases such as interstitial lung disease (ILD) rather than obstructive asthma. Like in ILD, there was limited post-bronchodilator reversibility. Atopic asthma is frequently associated with an atopic history and identifiable inhalant allergen sources. The patient presented at the onset of local tree pollination, however the SPT positivity that was only 1 mm larger than the saline control was inadequate to confirm pollen sensitization. Confirmatory serological tests were also negative at a cut off of < 0.35 kU/L. Tests with higher sensitivity (cut- off of < 0.1 kU/L) were not available. The absence of an atopic history, non-obstructive spirometry results and a failure to demonstrate allergen sensitization by both SPT and serology led us to conclude that sensitisation to inhalant allergen sources was unconvincing and allergic asthma was unlikely. The asthma-like symptoms appeared to be IgE independent and could have been non-allergic, irritant induced. Symptom resolution upon discontinuation of exposure to DEEE strengthened the suspicion that these had triggered the symptoms. The patient had twice tested weakly positive for the Ro-52 (TRIM-1) autoantibody. She had an elevated ESR and relative lymphocytopenia both of which are associated with connective tissue diseases. It is unclear whether the presence of the autoantibody in this patient was incidental, predictive or permissive of the manifestation of asthma-like symptoms.

### Health effects of exposure

The exposure to DEEEs in the fossil-fuel driven economy is ubiquitous and primarily through inhalation. The emissions contain particulate materials that are less than 2.5 microns in diameter (PM 2.5), that can evade mucociliary clearance and lodge in the bronchioles. The disease triggering mechanisms are unclear, immunological mechanisms have been proposed. Inhalation of the fumes affects lung development, causes respiratory disease and exacerbates pre-existing asthma, bronchitis or emphysema [14]. The highest risk of DEEE related adverse health effects is in truckers, bus depot operators and manned toll gate personnel. Commercial bank office workers are not recognized as being at risk. The health effects of exposure to DEEE are not trivial. Within the respiratory tract, they exacerbate pre-existing, predominantly house dust mite driven asthma, COPD or rhinitis. The inhalation of diesel engine fumes is implicated in myocardial ischaemia and thrombotic sequelae, [15] the development of childhood asthma [16] and lung cancer (IARC) [8]. Prompt diagnosis is imperative but requires appropriate human resource expertise and equipment that is often lacking in low income countries. The diagnosis of DEEE induced respiratory disease can also be delayed because it may be masked by or overlap with more common conditions such as atopic asthma. Awareness of the condition is low amongst non-occupational disease specialists who see a majority of asthma patients, it is rarely suspected and there are no diagnostic biomarkers. The increasing use of diesel generators in households and offices should prompt attending practitioners to include exposure to diesel fumes in the differential diagnoses of unexplained respiratory symptom exacerbations.

### Antinuclear autoantibodies and asthma-like symptoms

The repeated detection of anti-Ro-52 autoantibodies was intriguing. The autoantibodies that present in a range of autoimmune conditions and in healthy individuals are not considered disease specific. Although there was no overt autoimmune disease, a combination of a high ESR, reduced T lymphocytes and the detection of autoantibodies is usually associated with autoimmunity. The presence of antinuclear antibodies (ANA) but not specifically anti-Ro-52 was previously reported by Agache et al. who observed ANA to be strongly associated with severe asthma exacerbations, use of high doses of inhaled steroids and the risk of death [17]. Ferreira et al. have reported that the detection of Ro-52 autoantibody was “*very sensitive for ILD diagnosis*” [18]. ILD is also characterised by a restrictive spirometry pattern (low FVC, normal or increased FEV1/FVC ratio) as observed in this patient who had severe asthma-like symptoms and was refractory to high doses of inhaled steroids.

The relevance of anti-Ro-52 in this patient is unclear. Mechanistic explanations of the relationship between autoimmunity and asthma have eluded researchers. IL-17 cytokines produced by Th17 regulatory T lymphocytes have been previously shown to play a role in the pathogenesis of both asthma and autoimmune diseases. Dong and Brandt et al. reported that DEEE particles induced IL-17A expression thereby contributing to asthma severity [19, 20]. Evidence from murine [21] and human studies suggest that diesel particles exacerbate pre-existing lung disease via Th-17 related mechanisms, and can cause lung damage in the absence of prior allergic sensitization. Zhang et al. [22] studies of human nasal mucosa have identified DNA methylation sites that are associated with (a) asthma and allergy and (b) with responses to exposure to traffic air pollution in the absence of pre-existing asthma. The expression of genes in adjoining methylation sites increases upon exposure to either diesel emission particles or house dust mites. House dust mites and DEEE can independently exert their effect on bronchial mucosa through epigenetic mechanisms that alter the airway methylome and hydroxy methylome. Zhang et al. [23] the two agents DEEE and *D. pter*. also, independently regulate airway epithelial cell gene expression by altering the promoter or suppressor activities of histone modifiers. These studies, when taken together suggest that DEEE antigens, like *D. pter.* can induce asthma-like symptoms via identifiable immunological pathways that involve Th-17, methylation and epithelial gene upregulation [23] all of which can play a role in the development of asthma like symptoms following exposure to diesel engine exhaust fumes. DEEEs should thus be considered potential triggers of asthma symptoms regardless of pre-existing disease and could contribute to the development of ILD.

## Conclusion

Atypical presentations of adult onset asthma in the absence of a history of either atopy or allergen specific IgE antibody sensitization should trigger in-depth evaluation of occupational exposure in all cases including office workers. The early diagnosis of DEEE induced pulmonary dysfunction prevents lung damage and carcinogenesis. A diagnosis can be delayed by The absence of specialists, lack of relevant equipment (spirometers) and narrow descriptions of the at-risk population that now include a secretary in a financial sector institution can potentially delay the diagnosis.

## Abbreviations

ACT: Asthma Control Test
BMI: body mass index
DEEE: diesel engine exhaust emissions
IARC: International Agency for Research on Cancer
Ro52: Robert 52, a 52 kD antigen detected in the sera of some patients with connective tissue diseases
ILD: interstitial lung disease
SPT: skin prick testing
